# Quantification of particle-induced inflammatory stress response: a novel approach for toxicity testing of earth materials

**DOI:** 10.1186/1467-4866-13-4

**Published:** 2012-04-18

**Authors:** Andrea D Harrington, Stella E Tsirka, Martin AA Schoonen

**Affiliations:** 1 Department of Geosciences, Stony Brook University, Stony Brook, NY 11784-2100, USA; 2 Pharmacological Sciences, Stony Brook University, Stony Brook, NY 11794-8651, USA

## Abstract

**Background:**

Reactive oxygen species (ROS) are vital regulators of many cellular functions in the body. The intracellular ROS concentration is highly regulated by a balance between pro-oxidants and anti-oxidants. A chronic excess of pro-oxidants leads to elevated ROS concentrations and inflammation, possibly initiating or enhancing disease onset. Mineral-induced generation of ROS, the role of minerals in upregulating cellular ROS, and their role in the development of several occupational diseases are now widely recognized. However, there is no standard protocol to determine changes in ROS production in cells after exposure to mineral dust or earth materials in general. In this study, a new method for determining the degree of cellular toxicity (i.e., cytotoxicity) of particles is described that will help bridge the gap in knowledge.

**Results:**

By measuring the production of ROS and the viability of cells, an inflammatory stress response (ISR) indicator is defined. This approach normalizes the ROS upregulation with respect to the number of viable cells at the time of measurement. We conducted experiments on a series of minerals and soils that represent materials that are inert (i.e., glass beads, anatase, and a soil with low trace element content), moderately reactive (i.e., soil with high trace element content), and highly reactive (i.e., pyrite). Inert materials generated the lowest ISR, averaging 350% compared to the control. Acid washed pyrite produced the highest ISR (1,100 fold higher than the control). The measurements conducted as a function of time showed a complex response. Most materials showed an increase in ISR with particle loading.

**Conclusions:**

The amount of cellularly generated ROS and cell viability combined provide a better understanding of particle-induced oxidative stress. The results indicate that some earth materials may solicit an initial burst of ROS, followed by a second phase in which cell viability decreases and ROS production increases, leading to a high ISR value. Hence, measurements conducted over a range of particle loading combined with multiple data measurements up to 24 hours can provide new insights in the possible effect of exposure to earth materials on human health.

## Background

The inhalation of mineral, coal, and volcanic dust can lead to a spectrum of lung diseases [1-12]. Even in cases where the disease origin is clear (e.g., asbestosis, silicosis and coal workers' pneumoconiosis (CWP)), it is often unclear what role the earth material plays in the mechanism of pathogenesis (disease development). However, a common factor appears to be the upregulation of reactive oxygen species (ROS) in lung cells upon exposure [9,13-18]. ROS are short-lived intermediate reduction products of molecular oxygen: superoxide (O_2_· ^-^), hydrogen peroxide (H_2_O_2_), and hydroxyl radical (·OH) [19,20]. Although their formation is tightly regulated in the cells and tissues [21], the upregulation of ROS levels within lung cells as a result of an exposure to particulate matter can lead to a state of chronic inflammation. This inflammation is thought to pave the way for the development of lung disease [22,23]. The emphasis of most studies has been in the context of occupational health or extremely high particle exposures, such as in areas affected by deposition of volcanic ash [24]. However, there are growing concerns that chronic exposures to soil and mineral aerosols at particle loading levels much lower than those encountered in mines and other occupational environments where workers are exposed to high loading of particles can lead to public health issues [25-28]. Aerosol-induced upregulation of ROS appears to be a contributing factor in the health burden caused by these exposures with non-occupational particle loadings [25,27-29]. Given the possible critical role of particle-induced ROS upregulation upon inhalation, it is important to be able to measure this effect for a wide range of earth materials. Therefore, the aim of this study is to develop a method for determining the upregulation of ROS in epithelial lung cell cultures as a result of exposure to earth materials. The method is focused on epithelial cells because the lung lining is often where diseases such as silicosis, asbestosis, and CWP develop [30,31].

Earth materials can generate ROS spontaneously when dispersed in aqueous solutions free of cells and/or promote ROS upregulation in cells. It has been demonstrated that some minerals and other earth materials, such as coal, can spontaneously generate hydrogen peroxide and hydroxyl radical when dispersed in water [14,15,19,32-36] or simulated interstitial lung fluid [37-41]. Addition of earth materials to lung cell cultures can trigger an increase in ROS within the cells [42,43]. Importantly, materials that do not show formation of ROS when dispersed in a cell-free aqueous system may elicit an upregulation of ROS in epithelial cells as part of a cellular response to the presence of a foreign substance.

Two separate mechanisms are involved in the generation of ROS in cell-free mineral slurries. Surface defects may react with water or dissolved molecular oxygen to form radicals. This mechanism is thought to be important in the formation of ROS in quartz slurries. The quartz surface has several types of defects resulting from the homolytic and heterolytic cleavage of the Si-O bond. When exposed to water or molecular oxygen, these surface defects form ROS, see work by Hurowitz and coworkers and Fubine for details [13,19,35].

A second mechanism is based on the incomplete reduction of dissolved molecular oxygen through electron transfer reactions with a transition metal (made available via oxidative dissolution of particulates), most often ferrous iron [19]. The reactions relevant in the formation of ROS with ferrous iron are summarized below (Equations 1-4). The ferrous iron can either be aqueous ferrous iron or ferrous iron exposed on mineral surfaces.

(1) $$\text{F}{\text{e}}^{\text{2}+}+\;{\text{O}}_{\text{2}}\to \text{F}{\text{e}}^{\text{3}+}+\;{\text{O}}_{\text{2}}{\;}^{\cdot -}$$

(2) $$\text{F}{\text{e}}^{\text{2}+}+\;{{\text{O}}_{\text{2}}}^{\cdot -}+\text{ 2}{\text{H}}^{+}\to \text{F}{\text{e}}^{\text{3}+}+\;{\text{H}}_{\text{2}}{\text{O}}_{\text{2}}$$

(3) $${\text{H}}_{\text{2}}{\text{O}}_{\text{2}}+\text{ F}{\text{e}}^{\text{2}+}\to \text{F}{\text{e}}^{\text{3}+}+{\;}^{\cdot }\text{OH }{+}^{-}\text{OH}$$

(4) $$\text{3}{\text{H}}_{\text{2}}{\text{O}}_{\text{2}}+\text{ 2F}{\text{e}}^{\text{3}+}\to \text{2F}{\text{e}}^{\text{2}+}+\text{ 2}{\text{H}}_{\text{2}}\text{O }+\text{ 2}{\text{O}}_{\text{2}}+\text{ 2}{\text{H}}^{+}$$

Metals such as chromium, copper, vanadium, and manganese also generate ROS through the Fenton reaction (Equation 3) [44-46]. Although the reaction mechanisms for each of the Fenton metals has not been resolved at the same level of detail as with ferrous iron, the Fenton reaction (Equation 3) can be generalized to [47]:

(5) $${\text{H}}_{\text{2}}{\text{O}}_{\text{2}}+\;{\text{M}}^{\text{n}+}\to {\text{M}}^{\left(\text{n }+\text{ 1}\right)+}+{\;}^{\cdot }\text{OH }{+}^{-}\text{OH}$$

where M is a metal cation which can donate one electron and is stable at the higher oxidation state [47].

Epithelial lung cells exposed to particulate matter will upregulate ROS in an attempt to protect the cells against a foreign substance. Hydrogen peroxide release within epithelial lung cells is a non-specific defense mechanism designed primarily to kill pathogens [48], but the response is also triggered when cells encounter particulate matter [49]. Hence, the formation of ROS in cell-free aqueous systems is not a prerequisite for the upregulation of ROS when particles are added to lung cells, but particle-driven ROS formation may lead to enhanced ROS upregulation. The upregulation is expected to be particularly detrimental to cells if the material contains Fenton metals. Hydrogen peroxide produced in the cell will be converted to hydroxyl radical (Equation 5) [19], which will rapidly degrade a wide range of biomolecules leading to dysregulation of the cell and possibly cell death.

Cell death is one of the confounding factors in measuring the upregulation of ROS as a result of an exposure to particulate matter. Without correcting ROS measurements for cell viability, it is possible to incorrectly conclude that the exposure does not lead to ROS upregulation when, in fact, a significant fraction of the cells have died and those that survive have also been challenged and generate a higher ROS concentration. In the present study (see Experimental) we normalize the ROS readings with respect to the cell viability. We measure both ROS production in the cells and the cell viability using two separate assays. We utilize the A549 epithelial cell line, which is frequently used in toxicity studies [50-52]. It was first cultured in 1972 from the cancerous lung tissue of a 58 year old man. These adenocarcinoma human alveolar basal epithelial cells are easily cultured and proliferate quickly [53]. The relative "ease of use" and the fact that 90-95% of lung cancers are thought to originate in epithelial cells make this cell line an apt toxicity media for geoscientists [54,55].

The two assays used in this cellular study are both routinely used in the biomedical community. The cellularly-derived ROS concentration is determined using 2',7'-dichlorofluoroscein-diacetate (DCFH-DA). This molecule is added to the cell culture and is taken up by living cells. Once in the cell it is converted to DCFH. DCFH reacts with hydrogen peroxide as well as hydroxyl radicals to become fluorescent DCF [56]. The fluorescence associated with the formation of DCF is a measure of ROS production in the living cells. Due to the transient nature of ROS, it is important to use a probe that does not accumulate over time, but reflects both the rapid production and decay of ROS in the system. The cell viability is determined using 3-(4,5-dimethylthiazol-2-yl)-5-(3-carboxymethoxyphenyl)-2-(4-sulfophenyl)-2H-tetrazolium (MTS) [57], which produces a formazan product in the presence of phenazine methosulfate (PMS). The concentration of formazan product in the system is directly proportional to the number of living cells. This probe does not "predict" future apoptosis or necrosis but indicates the cell viability at the time of the assay measurement. Taken together, we define an indicator of overall "inflammatory stress response" (ISR) that is based on the ROS production normalized to cell viability (Equation 6).

(6) $$ISR=\frac{Cellularly\;Derived\;ROS}{Cell\;Viability}\;*\;\;100$$

Apart from using two routine biomedical assays in combination to define ISR, it is also important to use well characterized materials. In the development of this method, a range of natural and synthetic particulate matter was used as test materials. Anatase and silica glass, both synthetic, are often used as control materials to register the minimal cellular response to exposure to particulate matter because these materials are considered to have minimal reactivity. Two readily available reference soils from the National Institute of Science and Technology (NIST) with different metal concentrations (San Joaquin Valley NIST # 2709 and Montana NIST# 2710, see Table 1) were used. The San Joaquin Valley soil sample was collected after removing the top 13 cm of soil in a plowed field in the Panoche fan between the Panoche and Cantu creek beds in the San Joaquin Valley. This soil represents a natural material of low reactivity. The Montana soil was collected from the top 10 cm of soil along the Silver Bow Creek in Butte, which is located near a major copper mine. When the creek floods, high concentrations of copper, manganese, and zinc are deposited at the collection site. Given its elevated trace metal content, including Fenton elements (manganese and copper), this soil sample represents a moderately reactive material. In order to determine an upper range of ISR, the mineral pyrite is examined. The treatment of pyrite with acid removes the oxidized iron from the surface, allowing for more rapid ROS formation due to Fenton chemistry. The toxicity of these materials is most easily compared if the measurements are normalized with respect to exposed surface area [58-60]. This requires that the specific surface area of the material is determined and experiments are conducted over a range of particle loadings. Furthermore, it is useful to conduct measurements as a time series to capture the development of ROS over a period of 24 hours.

**Table 1:** Certified NIST elemental analysis of standard reference material [61,62]

Element	San Joaquin Valley Soil	Montana Soil
	Mass Fraction (%)	
	
Aluminum	7.50 ± 0.06	6.44 ± 0.08
Calcium	1.89 ± 0.05	1.25 ± 0.03
Iron	3.50 ± 0.11	3.38 ± 0.10
Magnesium	1.51 ± 0.05	0.853 ± 0.042
Phosphorus	0.062 ± 0.005	0.106 ± 0.015
Potassium	2.03 ± 0.06	2.11 ± 0.11
Silicon	29.66 ± 0.23	28.97 ± 0.18
Sodium	1.16 ± 0.03	1.14 ± 0.06
Sulfur	0.089 ± 0.002	0.240 ± 0.006
Titanium	0.342 ± 0.024	0.283 ± 0.010

	Mass Fraction (μg/g)	

Arsenic	17.7 ± 0.8	626 ± 38
Cadmium	0.38 ± 0.01	21.8 ± 0.2
Copper	34.6 ± 0.7	2950 ± 130
Lead	18.9 ± 0.5	5532 ± 80
Manganese	538 ± 17	22,124 ± 876
Mercury	1.40 ± 0.08	32.6 ± 1.8
Zinc	106 ± 3	6952 ± 91

## Experimental

### Culturing and Plating the A549 Human Lung Epithelial Cell Line

The A549 human lung epithelial cell line was cultured using Ham's F12K Media containing 10% Fetal Bovine Serum (FBS) and 1% 1X Penicillin/Streptomycin (A549 cell growth media). Once confluence was reached in T75 flasks (cells covering entire bottom surface), the cells were passaged (removed from original flask and placed in another to grow in fresh media) at 1 × 10^5 ^cells/mL using trypsin with Ethylenediaminetetraacetic acid (EDTA) to lyse the cells (remove from flask walls). For counting purposes, some of the cells were stained with Trypan blue and quantified using a hemocytometer. Wells in Columns 3-10 of the 96-well microplates were loaded with 8 × 10^4 ^cells/mL, covered with microplate lid, and allowed to incubate at 37°C in the A549 cell growth media until confluent (approximately 2 days). Due to background generated by the assays, Columns 1-2 and 11-12 were kept cell free for normalization. All work with cells took place in a sterile hood to avoid airborne bacteria that would affect the results. When not in the hood, the microplate lid was kept on at all times. The incubator provided a controlled environment in terms of temperature, relative humidity and carbon dioxide (CO_2_) concentration in air (37°C, 95% and 5% CO_2 _in air, respectively).

### Inflammatory stress response measurements

**Figure 1:**
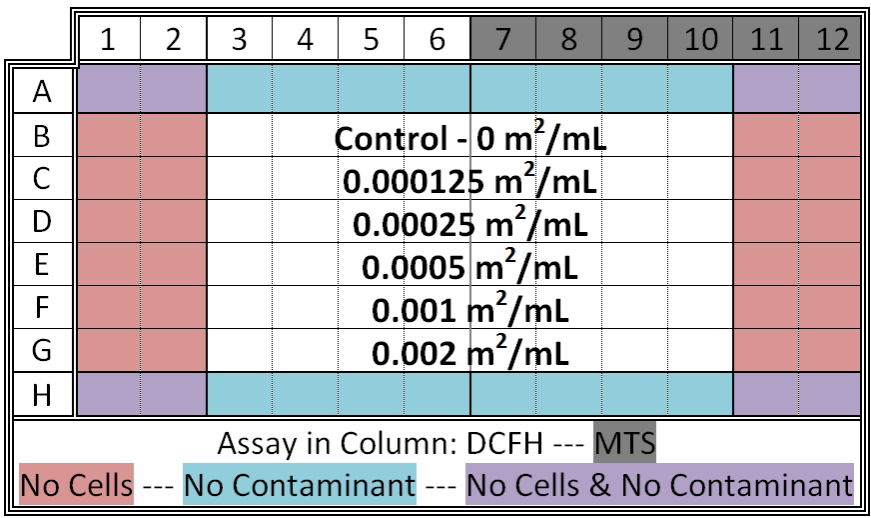
**Schematic of 96-well microplate for cellular study**. The arrangement for all microplates in this study is shown. The positioning of control wells, assay distribution and particle loadings are highlighted. For each material tested two identical plates were evaluated at the same time. Hence, for a given mineral and loading, the ROS level and cell viability was determined in 8 replicate wells.

After a two day incubation period, the cell growth medium was discarded by tipping the microplates into a container lined with sterile gauze, while the cells remained attached to the bottom of the plate. All liquid contents were allowed to drain out (approximately two minutes). Columns 1-6 were filled with 200 μL 50 μM DCFH-DA (from Sigma Aldrich) in Hank's Buffered Salt Solution (HBSS) and columns 7-12 were filled with 200 μL HBSS, and then placed in the incubator. During incubation, varying concentrations of the particle/contaminant slurry were created (normalized to surface area) via serial dilutions of a stock solution (i.e., 0.002 m^2 ^mineral/mL HBSS). After incubating for 20 minutes, all liquid contents of the microplate (DCFH-DA assay and HBSS) were again discarded (using the same approach as above). 200 μL of the contaminant slurry was added to the plates as shown in Figure 1. Rows A and H were kept free of contaminants due to edge effects, which simply put are widespread factors that cause the degradation of assays during high-throughput screening experiments [63]. Row B was kept free of particles for normalization (control). After slurry addition, 20 μL MTS (from Promega) was added to all wells in columns 7-12. The plate was then placed in the incubator for 20 minutes before the first analysis. Therefore, each microplate had samples for the ROS and the viability assays, five particle loadings, and control wells (Figure 1). For each material tested, two microplates with identical loadings and layouts were evaluated simultaneously. Hence, the data for each particle loading for a given material was the average of eight separate wells, four per microplate.

DCFH-DA is a fluorometric probe used to determine cellularly derived ROS and was analyzed using Thermo Scientific's Fluoroskan Ascent. MTS is a colorometric probe used to determine cell viability and was analyzed using Molecular Devices' SpectraMax 340PC384. The microplates were kept in the incubator at all times other than for analyses. During periods before analysis, microplates were kept in dark using foil. Plates were analyzed at 20 minutes, 40 minutes, 1 hour, 2 hours, 3 hours and 24 hours (upper limit of MTS usability [64]).

To ensure the pH of the slurries remained within a biologically applicable range, measurements were taken in separate, replicate experimental microplates. A Sensorex Combination pH Electrode (1/2 inch in diameter) attached to a Fisher Scientific Accumet Portable pH meter was inserted into various wells throughout the duration of experiment. Each well was measured once. The pH measurements are estimated to have an uncertainty of 0.1 pH units.

### Mineral sample preparation and soil standards

All specific surface areas were obtained using a Quantachrome NOVA 5-point BET analyzer using UHP N_2 _gas. The silica glass beads obtained from Corpuscular had a specific surface area of 0.326 m^2^/g. The mineral anatase (TiO_2_) was procured in powder form from the Aldrich Chemical Company with a specific surface area of 9.471 m^2^/g. Soil standards #2709 (San Joaquin Valley) and #2710 (Montana), acquired from NIST, had a surface area of 12.284 m^2^/g and 9.976 m^2^/g, respectively. The only material treated after procurement was the Huanzala pyrite, which was ground and sieved to a size below 38 μm. A portion of the ground pyrite was acid treated using a 0.1 M HCl solution to remove iron (oxy)hydroxide weathering products from the surface and stored under vacuum in an anaerobic glovebag. The specific surface areas of the untreated and acid washed pyrite were 1.887 m^2^/g and 1.923 m^2^/g, respectively. The estimated error in the BET measurements is 2.5%. This estimate is based on repeated analyses of a TiO_2 _surface area standard provided by Quantachrome.

## Results

**Figure 2:**
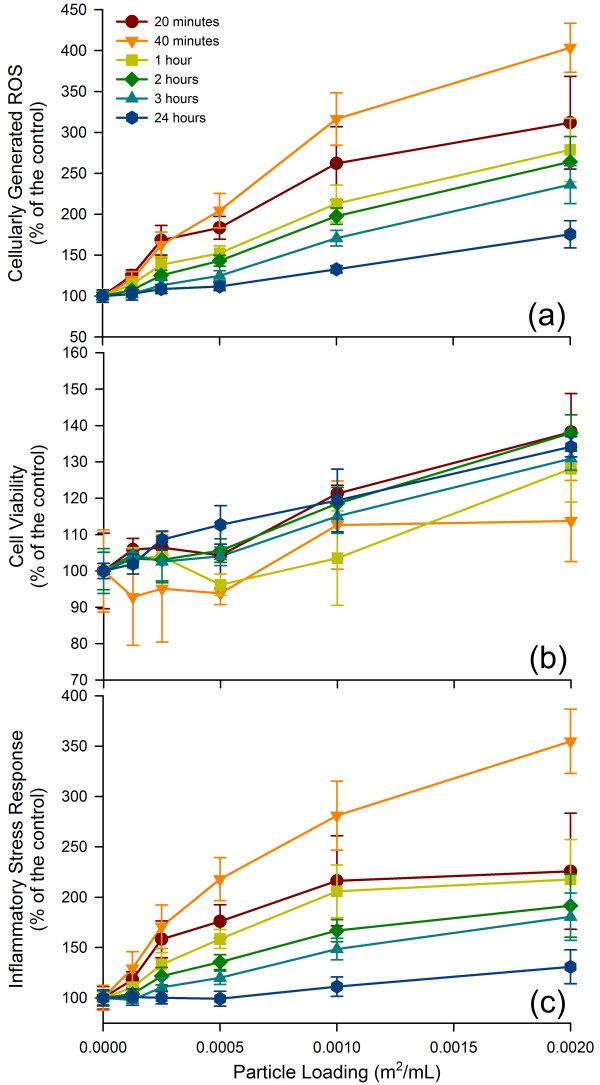
**ROS upregulation, cell viability and ISR of A549 cells to glass beads**. The cellular response to glass with increasing particle loading is presented with three different figures, all normalized to a control. (a) ROS upregulation, (b) cell viability, and (c) ISR - ROS upregulation divided by cell viability. For some data points, the error bars are hidden by the symbols.

Particle-derived ISR was determined for six materials with varying surface reactivities. Figure 2 illustrates the ROS production, the cell viability, and the calculated ISR for the exposure to glass beads. The error bars are calculated ± standard deviations based on the eight replicate measurements for ROS and cell viability (i.e., 4 identical conditions per microplate, two microplates in total), with the errors for the ISR calculated using a standard propagation method. Note that in the case of the glass beads the cell viability increased somewhat with loading, which led to a leveling off of the ISR curve as function of loading. Overall, it is clear that adding more glass beads resulted in an increase in ISR.

**Figure 3:**
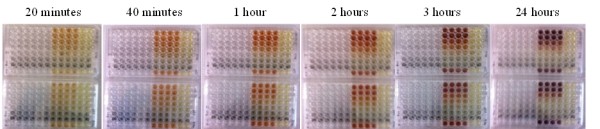
**Microplate evolution over time**. Microplate image displays the cellular response to acid treated pyrite over the course of the experiment. Two identical microplates are shown. The layout of the microplates follows the experimental designed presented in Figure 1. The changes in color on the right side of the microplate is due to development of the assay and becomes darker as cell viability/cell numbers increase. The lack of color in the wells with the highest amount of pyrite indicates low cell viability.

Figure 3 shows images of two microplates in which the A549 cells were challenged with acid-washed pyrite. The two plates had identical loadings and layout. The color changes developed over time on the right side of the microplate indicate changes in cell viability, which was determined using the MTS protocol. The intensity of the purple color increased over time as cell viability increased. The lack of the purple color indicates cell death.

**Figure 4:**
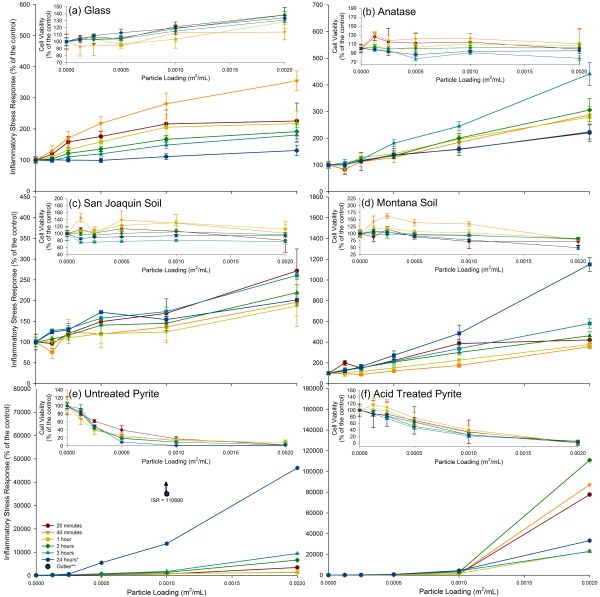
**ISR of six materials with varying surface reactivities**. The ISR of the six study materials are represented, with the cell viability for each in the inset. (a) Glass, (b) anatase, (c) San Joaquin Valley soil, (d) Montana soil, (e) untreated pyrite, and (f) acid treated pyrite. For some data points, the error bars are hidden by the symbols. * See Additional files 1 and Additional file 2 for clarification.

**Figure 5:**
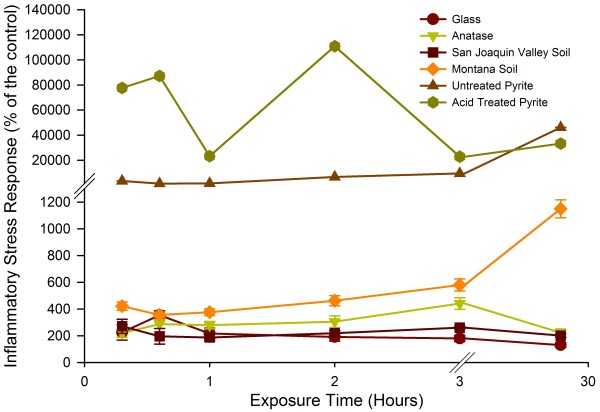
**Evolution of ISR over time**. The ISR over time of all the materials at the highest particle loading (0.002 m^2^/mL) is represented. For some data points, the error bars are hidden by the symbols.

The ISR and cell viability for all materials tested in this study are shown in Figure 4. In each panel in Figure 4, the cell viability data are shown as an inset. The ISR over time for all the materials tested is represented in Figure 5. In separate replicate experiments, we determined that the addition of the earth materials itself did not lead to a significant drop in pH within the cell cultures. The cell cultures challenged with the highest loadings of pyrite, expected to be the most reactive material in terms of changing slurry pH, did not deviate from the pH in the control by more than 0.3 pH units.

Anatase and glass beads are relatively inert particulates and these materials are often used in toxicity studies to represent base level responses [65,66]. These two materials, as well as the San Joaquin Valley soil, showed no drastic decrease in cell viability. The ISR generally increased with particle loading at any given time point for these three materials. Their presence generated up to 450% (anatase) and 350% (glass beads) the ISR compared to measurement of ISR in cells without any particles added (control). The San Joaquin Valley soil generated a lower ISR (250% at highest loading compared to control). While all three materials are relatively inert, the ISR over time was different for each material. The San Joaquin Valley soil generated the highest ISR within the first 20 minutes, the glass after 40 minutes and the anatase after 3 hours (Figure 5). The ISR in the glass beads and anatase experiments showed a progression toward the maximum levels of quantified stress and then a decrease over time. The data for the San Joaquin Valley soil showed a more erratic pattern with the ISR measured after 20 minutes and 3 hours nearly the same, while the measurements after 40 minutes and 1, 2 hours were lower. As shown in Figure 4c, the error bars for several of the data sets overlap. Hence, it is not possible to extract definitive trends for ISR as a function of time that are representative of the whole "inert" group of materials tested here.

The Montana soil, which is a soil impacted by mining activity that contains high levels of transition metals, caused a significant drop in cell viability over a period of 24 hours (60% viability) along with a significant ROS signal. As a result, the ISR caused by the Montana soil was by a factor of 11 above the control after 24 hours at the highest loading. The Montana soil showed a spike in ISR after 20 minutes, just as the San Joaquin Valley soil, followed by a drop in ISR. A systematic increase in ISR was then measured from 40 minutes to 24 hours after the start of the experiment. This suggests that there was both an acute response that led to elevated ISR, followed by a delayed response that led to elevated ISR. The initial cell response to the insult event generated a burst of ROS, possibly to try and rid the area of foreign substances. A slower process then took over, potentially indicative of cellular adaptation to the stress inducer, and generated a relatively low ISR over the next 21 hours.

The ISR generated by both the treated and untreated pyrite was at least a factor of 40 higher than the peak ISR of the Montana soil and a factor of 100 higher than the peak for anatase. The untreated, or oxidized, pyrite generated an ISR value that was 450-fold higher than the cells without particles after 24 hours. The cell viability was also immediately affected by the presence of this pyrite. At the highest pyrite loading the cell viability is reduced to 1%. At the highest loading, the acid washed pyrite generated an ISR value that was 1,100-fold the control after 2 hours. It is important to note the variation in ISR over time of the two pyrite samples. After 2 hours, the ISR generated by oxidized pyrite was 20% of the level seen at 24 hours. Hence, the ISR in the experiment with oxidized pyrite increased over time. By contrast, the ISR measured in the experiment with acid washed pyrite decreased by a factor of four between the 2 hour and 24 hour time points for the highest particle loading.

The data for the untreated pyrite after 24 hours contained an anomalously high reading for the 0.001 m^2^/mL loading. Labeled as an outlier on Figure 4e, this data point, which is the average of eight separate wells with identical conditions over two microplates, yielded extremely low cell viability readings. When the cell viability values of two the surrounding particle loadings (0.0005 m^2^/mL and 0.002 m^2^/mL) were used to estimate the cell viability for the 0.001 m^2^/mL loading using a weighted average approach, the calculated ISR value was adjusted downward and falls on a curve that shows the expected general increase in ISR with particle loading (See Additional file 1 and Additional file 2). This particular data point underlines the importance of including three separate factors in the experiments. One factor is abundant replication, which is here made possible by using duplicate 96-well plates, leading to eight separate measurements for each condition. The second factor is that the 96-well plates make it possible to experiment with multiple loadings. The last factor is that multiple measurements are made over time allowing for trends to emerge that show an increase in ISR with increased particle loading. Any major deviation from a general increase of ISR with increased particle loading requires a closer examination of both the ROS upregulation and cell viability data.

## Discussion

Utilizing both cell viability and cellular ROS generation assays, a protocol is created for determining ISR induced by earth materials. By measuring the ROS production and cell viability within minutes to hours for a period up to 24 hours, the method provides insight into the development of ISR over time. The dose-response is evaluated by varying the particle loading. For highly reactive particles, it is important to adjust the particle loading so that cell viability remains measurable, as cell viability is the denominator in the ISR term.

All materials tested here show a general increase in ISR with increasing particle loading or dose; however, the development of ISR over time is different for each material. As illustrated by the results for the Montana soil (Figure 4d), an initial burst of ROS formation immediately upon addition of the particles may be followed by a separate, slower process that leads to upregulation of ROS. The standard for toxicity studies is for plates to be analyzed at one time point (commonly 3 hours or 24 hours) after particulate exposure [67-69]. If this approach was used and the plates were only read at 3 hours, then the ISR for both the oxidized pyrite and acid washed pyrite would be underestimated by 500%. If the plates were only read at 24 hours, then the ISR for the acid washed pyrite would be underestimated by 330%. Due to limitation of the stability of the assays the experiments were terminated at 24 hours [64]. However, in the case of the materials that generate an increasing ISR with time, the ISR is expected to continue to increase. In fact, an ISR is expected to persist until complete particle dissolution. Therefore, the ISR measurements represented here serve as an indication of possible long-term toxicity.

For this new protocol, experiments were conducted on six materials that offer a wide spectrum of responses. The glass beads and anatase showed little effect on cell viability but caused some upregulation of ROS. Although often used to represent baseline stress levels, anatase does generate a measureable amount of ROS [70,71]. This upregulation likely represents the minimum cellular response to the presence of an otherwise unreactive foreign material. This low ISR was also observed with the San Joaquin Valley soil, which is characterized by NIST as a soil with baseline trace element concentrations. By comparison, the Montana soil is enriched in metals, including possible Fenton metals other than iron (e.g., Cu, Mn, see Table 1) as well as toxic metals that may affect cell viability (e.g., Pb, Zn, As, Cd, see Table 1). The high metal content in the Montana soil is likely the cause for the higher ISR value, which is due to a combination of cell death and ROS upregulation in the remaining cells.

Pyrite elicited by far the strongest ISR. In earlier work we showed that pyrite is capable of producing ROS when dispersed in water or simulated lung fluid [41]. We speculate that when epithelial cells are exposed to pyrite they form hydrogen peroxide as part of the normal immune defense response. However, the presence of ferrous iron promotes the Fenton reaction, which leads to the formation of hydroxyl radical. The production of hydroxyl radical within the cells is likely the cause for the high degree of cell death in the experiments with pyrite. The surviving cells are compromised and as a result of the normalization with respect to cell viability the ISR rises to very high levels. The difference in the development of the response to acid-washed pyrite and oxidized pyrite is likely due to the presence/absence of ferric oxide patches on the surface. As shown in earlier work [72], the presence of ferric oxide patches on the surface of pyrite leads to the decomposition of hydrogen peroxide to water and molecular oxygen (Equation 4), rather than hydroxyl radical. However, over time dissolved ferrous iron does build up and the Fenton reaction goes forward (Equation 3). Acid washing of pyrite removes any ferric oxide patches [73,74]; hence, acid-washed pyrite is expected to very effectively promote the formation of hydroxyl radical in a cell that is producing hydrogen peroxide until the surface become partially covered with ferric oxide patches.

From an occupational health perspective, mine workers who create fresh rock dust containing pyrite are likely to inhale pyrite that would stimulate a burst of hydroxyl radical formation. On the other hand, settlements downwind from pyrite-containing mine tailings might be inhaling dust with oxidized pyrite. Both exposures lead to inflammation, but the exposure to fresh pyrite surfaces will lead to higher levels of inflammation. Furthermore, the data for untreated pyrite data suggest that the ISR would continue to increase with time. Given the form pyrite takes in coal, complete dissolution of a single framboidal pyrite particle would occur within a year [41,75]. It is unknown whether a short-lived spike in ISR is more important to pathogenesis than a lower more prolonged ISR. However, it must be taken into consideration that these experiments represent a single exposure event. In the case of a coal miner, each inhalation could be exposing the miner to reactive materials. The average adult takes between 12-20 breaths per minutes, so average the coal miner would have approximately 2,000,000 exposures in a year (assuming a 40 hour work week).

Overall the protocol presented here can be used to characterize a wide range of earth materials in terms of their ability to induce an ISR in cells. Complemented by acellular assays to determine particle-driven ROS formation, chemical analysis, and a well-established provenance of the sample, the protocol presented here can provide a rapid assessment of the ability of the earth material to provoke upregulation of ROS, lead to cell death, and induce inflammation in the remaining cells. The materials tested here are only a small subset of the materials that are of interest from a public health or occupational health perspective.

## Conclusions

The method described here is designed to test the ability of earth materials to cause cell death and upregulate ROS in cells. Both dose-response and temporal changes in ISR can be resolved by the protocol. The method provides the geosciences community an opportunity to contribute to the emerging field of medical geology by combining two well-established assays. Using the method described here along with knowledge of the provenance, composition and treatment of earth materials is expected to lead to new insights into the effect of earth materials on public and occupational health.

## Abbreviations

(ISR): Inflammatory stress response; (DCFH-DA): 2',7'-dichlorofluoroscein-diacetate; (MTS): 3-(4,5-dimethylthiazol-2-yl)-5-(3-carboxymethoxyphenyl)-2-(4-sulfophenyl)-2H-tetrazolium; (FBS): Fetal bovine serum; (HBSS): Hank's Buffered Salt Solution; (EDTA): Ethylenediaminetetraacetic acid; (CWP): Coal Workers' Pneumoconiosis; (ROS): Reactive oxygen species; (·OH): Hydroxyl radical; (H_2_O_2_): Hydrogen peroxide; (NIST): National Institute of Standards and Technology.

## Competing interests

The authors declare that they have no competing interests.

## Authors' contributions

ADH helped design the study, performed the experiments, and drafted the manuscript. SET and MAAS helped design the experiments, supervised the study, and edited the manuscript. All authors have read and approved the final manuscript.

## Supplementary Material

Additional file 1 **Contains information detailing the correction technique, as well as the justification, used for the 24 hour time point for Untreated Pyrite with a loading of 0.001 m^2^/mL**. Click here for file

Additional file 2 **Is a figure that complements Additional file **1**by demonstrating the accurate projection of ISR for the 0.001 m^2^/mL using a weighted average**. Click here for file
