# Supplementary material for: Quantification of particle-induced inflammatory stress response: a novel approach for toxicity testing of earth materials
Source: Geochem Trans. 2012 Apr 18;13:4. doi: 10.1186/1467-4866-13-4 (PMC3351022; doi:10.1186/1467-4866-13-4)
Supplement: Additional file 1 — Contains information detailing the correction technique, as well as the justification, used for the 24 hour time point for Untreated Pyrite with a loading of 0.001 m2/mL. [file 1467-4866-13-4-S1.DOCX]

**Additional File 1.**

The method for determining an inflammatory stress response (ISR) requires the upregulation of reactive oxygen species (ROS) to be divided by cell viability. This normalization is important when determining the amount of cellular burden. However, when cell viability values are low, errors in cell viability values can lead to an erroneously inflated ISR value. This issue occurs with the untreated pyrite 24-hour data point at 0.001 m^2^/mL (Figure 4e). Although there is no visual anomaly with the individual plate wells, only 0.7 % of the cells are still viable when compared to the control. This viability value is more than an order of magnitude lower than the viability value for the sample with 0.0005 m^2^/mL loading (9.5% viability compared to the control). Furthermore, the cell viability in the 24-hour data point at 0.001 m^2^/mL is close to half of what is measured for the sample with the 0.002 m^2^/mL loading (1.2% viability compared to the control), which is counter to the notion that a higher particle loading is expected to lead to lower cell viability. In short, the cell viability of the 24-hour data point at 0.001 m^2^/mL appears to represent an anomalous value. Given that it is expected that cell viability is correlated with loading, we have evaluated this relationship and used it to estimate what the expected cell viability of the 24-hour data point at 0.001 m^2^/mL should be. The calculated value is based on the measured cell viabilities of the 24-hour data points for the 0.0005 and 0.002 m^2^/mL conditions.

The notion of calculating cell viability/numbers for a given treatment on the basis of measured viabilities is illustrated with data for anatase (a relatively inert material showing limited cell death) and acid treated pyrite (a highly reactive material showing large amounts of cell death). Specifically, the calculated cell viabilities for the 0.001 m^2^/mL are weighted averages of cell viability values measured at the next higher loaded (0.002 m^2^/mL) and the adjacent lower loading (0.0005 m^2^/mL). The viability of the cells at the 0.001 m^2^/mL loading is calculated using the following equation:

$$\frac{A+1.5B}{2.5}=C$$

Where A is the viability at 0.0005 m^2^/mL; B is the viability at 0.002 m^2^/mL; and C is the viability at 0.001 m^2^/mL. Calculated and measured cell viability values are compared in Figure A1. The calculated cell viability at the 0.001 m^2^/mL loading for acid treated pyrite and anatase compare favorably to the actual measurements. On the basis of this relationship the viability of the cells in the untreated pyrite experiment at 0.001 m^2^/mL is estimated to be 4.6% compared to the control. When the estimated cell viability for this data point is used, the resulting ISR value falls on a dose-response curve that is consistent with the notion that higher particle loading is expected to lead to higher ISR.

**Figure Legends**

**Figure A1 – Measured and calculated ISR over time**

The measured and calculated ISR over time for anatase, untreated pyrite, and acid treated pyrite at the 0.001 m^2^/mL particle loading is demonstrated.
