# Supplementary figures and images for: Quantification of particle-induced inflammatory stress response: a novel approach for toxicity testing of earth materials
Source: Geochem Trans. 2012 Apr 18;13:4. doi: 10.1186/1467-4866-13-4 (PMC3351022; doi:10.1186/1467-4866-13-4)

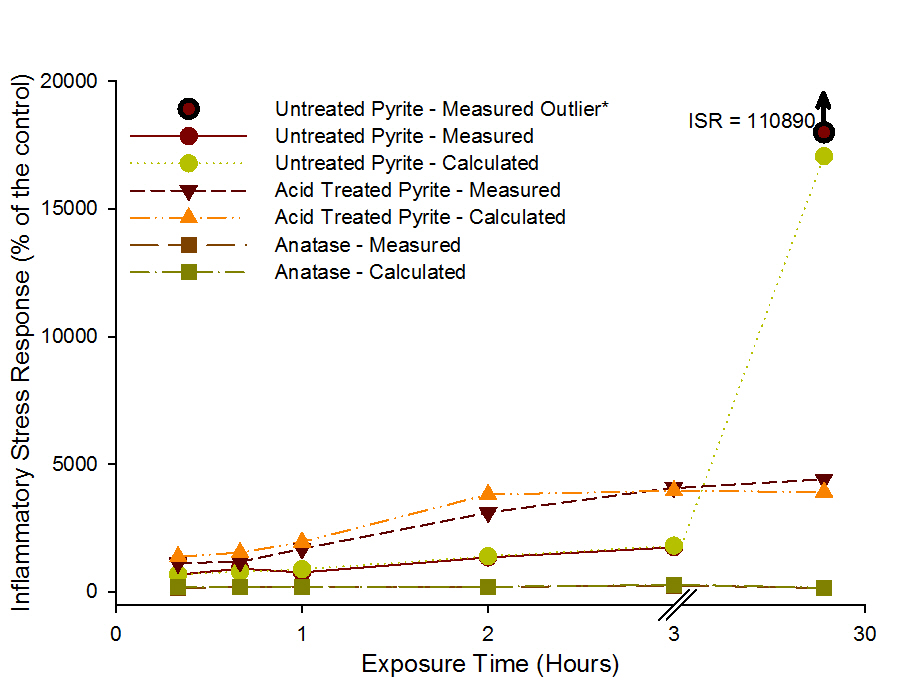

Supplement: Additional file 2 — Is a figure that complements Additional file 1by demonstrating the accurate projection of ISR for the 0.001 m2/mL using a weighted average. [file 1467-4866-13-4-S2.JPEG]
